# What Predicts Adolescent Delinquent Behavior in Hong Kong? A Longitudinal Study of Personal and Family Factors

**DOI:** 10.1007/s11205-015-1170-8

**Published:** 2015-11-13

**Authors:** Daniel T. L. Shek, Li Lin

**Affiliations:** Department of Applied Social Sciences, The Hong Kong Polytechnic University, Hunghom, Kowloon, Hong Kong

**Keywords:** Delinquent behavior, Economic disadvantage, Family intactness, Family functioning, Positive youth development, Chinese adolescents

## Abstract

Using four waves of data from Secondary 1 to Secondary 4 (*N* = 3328 students at Wave 1), this study examined the development of delinquent behavior and its relationships with economic disadvantage, family non-intactness, family quality of life (i.e., family functioning) and personal well-being (i.e., positive youth development) among Hong Kong adolescents. Individual growth curve models revealed that delinquent behavior increased during this period, and adolescents living in non-intact families (vs. intact families) reported higher initial levels of delinquent behavior while those living in poor families (vs. non-poor families) showed a greater increase in delinquent behavior. In addition, with the demographic factors controlled, the initial levels of family quality of life and personal well-being were negatively associated with the initial level of delinquent behavior, but positively associated with the growth rate of delinquent behavior. Regression analyses showed that family quality of life and personal well-being were related to the overall delinquent behavior concurrently at Wave 4. However, Wave 1 family quality of life and personal well-being did not predict Wave 4 delinquent behavior with the initial level of delinquent behavior controlled. Lastly, we discussed the role of economic disadvantage and family non-intactness as risk factors and family functioning and positive youth development as protective well-being factors in the development of adolescent well-being indexed by delinquent behavior.

## Introduction

Delinquent behavior increases during adolescence, especially early adolescence, in both the Western (e.g., Farrell et al. 2005; Overbeek et al. 2001) and Chinese contexts (Shek and Yu 2012; Shek and Lin 2014a). Many studies have been conducted to identify risk and protective factors of delinquent behavior during adolescence (e.g., Jessor et al. 2003; Jessor and Turbin 2014). For the risk factors that enhance the likelihood of delinquent involvement, family adversity in terms of economic disadvantage (see McLoyd et al. 2009 for a review) and family disruption (see Lansford 2009 for a review) is strongly emphasized in the previous literature. Although many studies have examined the association between family adversity and delinquent behavior at a single point, few studies have looked at whether economic disadvantage and family disruption influence the developmental trajectory of delinquent behavior over the adolescent years. For the protective factors that lower the likelihood of delinquent involvement, family functioning (Schwartz et al. 2005; Shek 2002b) and positive youth development (Benson et al. 2006; Geldhof et al. 2014; Sun and Shek 2010, 2012) have been proposed to be the relevant family and personal well-being factors. Nevertheless, longitudinal research examining their long-term effects on delinquent behavior, especially in non-Western contexts, is still lacking.

As suggested by Bongers et al. (2004), investigation of delinquent behavior at any single point during adolescence may limit our understanding of the phenomena. Therefore, we investigated the risk factors (i.e., economic disadvantage and family non-intactness) and protective factors (i.e., family functioning and positive youth development) using four waves of data from a large sample of Hong Kong Chinese adolescents in this study. First, we examined how these risk factors and protective factors were related to the initial level (Wave 1) and developmental trajectory of delinquent behavior across four waves. Next, we examined how these factors concurrently (Wave 4) and longitudinally predicted delinquent behavior (Wave 1 factors predicting Wave 4 delinquent behavior).

### Developmental Trajectory of Delinquent Behavior

The developmental perspective of delinquent behavior concerns the question of how delinquent behavior develops as a function of time or age. It appears to be a consensus that a notable increase occurs when children transit into adolescence, while a decline occurs when they transit to adulthood (e.g., Deković et al. 2004; Overbeek et al. 2001). For example, based on parent-reports of 1302 adolescents, Overbeek et al. (2001)’s study showed that early adolescents had lower levels of delinquent behavior than mid-adolescents, while late adolescents had higher levels than adults. Longitudinal assessment of different cohorts of adolescents provides even more convincing evidence. For example, Stanger et al. (1997) examined parent-report delinquent behavior of seven birth cohorts of Dutch children across five times at 2-year intervals and found support that delinquent behavior boosts during adolescence.

Compared to the abundance of research in Western adolescents, research is unfortunately limited in Chinese adolescents who, however, account for over 14.8 % of the world total population (World Health Organization 2010). Jessor et al. (2003)’s study comparing mainland China with the United States showed that Chinese adolescents demonstrated lower levels of problem behavior (i.e., problem drinking, cigarette smoking, and general delinquency). However, with a single wave of assessment, the findings were unable to portray the developmental course of delinquent behavior. In Hong Kong, a pioneer study has tracked the developmental trend of delinquent behavior over 5 years of secondary school across eight waves of assessment (Shek and Yu 2012). An increasing trend was observed from Secondary 1 to Secondary 5. Nonetheless, there is a dearth of study examining any individual differences in the rate of change of delinquent behavior. Specifically, it is still unclear whether risk factors that enhance delinquent engagement will be associated with faster increase of delinquent engagement and protective factors that reduce delinquent level will be associated with slower increase during adolescence.

### Risk Factors: Economic Disadvantage and Family Non-intactness

Among the risk factors that account for the heightened level of delinquent behavior, two of them are of our central interest. One is economic disadvantage and the other is family non-intactness. Experiencing poverty is linked to an increased likelihood that adolescents will show delinquent behavior (Mcloyd 1998; Mcloyd et al. 2009). From the social control perspective (Hirschi 1969), poor families may not be able to provide sufficient resource, experience, and support constructive for shaping social bonding of adolescents (e.g., parent–child bonding; school bonding), whereas good bonding implies adherence to conventional rules and values. Therefore, adolescents from poor families might demonstrate more norm-breaking or antisocial acts than do those from families without economic difficulties. From the family stress perspective (Conger and Conger 2008), economic hardship arouses parents’ stress, which dampens their well-being, marital relationship and eventually effective parenting. The maladaptive family dynamics, especially disruptive parenting, further puts poor adolescents at greater risk of delinquent involvement.

However, the studies that focused on how poverty was associated with the level of delinquent behavior yield discordant results (Mcloyd et al. 2009). Pagani et al. (1999)’s study found a direct link between poverty (e.g., low family income) and delinquency in 16-year-old boys. Yet such direct association was not significant in many other studies (e.g., Conger et al. 1994; Gonzales et al. 2011). Shek and Lin (2014b)’s study in a Hong Kong sample revealed little difference in delinquent behavior between adolescents from the families that were receiving government welfare due to economic hardship and adolescents from the families without receipt of government welfare.

Do such non-significant associations indicate that economic disadvantage has no impact on adolescent delinquent behavior? This conclusion may be too hasty without probing into how economic disadvantage affects the developmental course of delinquent behavior. The boost of delinquent behavior during adolescence probably relates to adolescents’ transitional challenges in diverse domains (i.e., physical, cognitive, socio-emotional domains). Poverty presumably exacerbates the challenge to cope with difficulties among adolescents whose families are unable to offer them with relevant resource, experience and service (Murry et al. 2011). Consequently, poor adolescents might increase their delinquent involvement faster than non-poor adolescents. Unfortunately, compared with the heated discussion on the relationship between economic disadvantage and the level of adolescent delinquent behavior, scanty research has examined its relationship with the developmental trajectory of delinquent behavior.

In addition to economic disadvantage, family non-intactness is also a potential risk factor for juvenile delinquency. Compared with intact families (i.e., living with two biological parents), adolescents from non-intact families (i.e., at least one biological parent is missing) often demonstrated higher levels of delinquent behavior (e.g., Jolliffe 2013; Shek and Leung 2013; Vanassche et al. 2014). Lack of physical and psychological parental control may be one of the primary factors leading to this observation (Demuth and Brown 2004; Rebellon 2002). Parents in non-intact families are less likely to provide adequate parental control and supervision or set up appropriate rules and regulations, possibly due to their high involvement in working for a living or their distress resulting from marital failure and livelihood difficulties. Because of insufficient parental involvement, parent–child closeness might be weakened in non-intact families, while intimate parent–child relationship serves as an indirect control. Adolescents having a close relationship with their parents will care about parents’ expectation on adherence to conventional rules, which prevents adolescents from delinquent involvement (Hirschi 1969; Hoeve et al. 2012). In addition, exposure to inter-parental conflict in non-intact families may also put adolescents at risk (Lansford 2009). In particular, a hostile family environment with overt conflicts may drive adolescents associated with delinquent peer, from whom they learn to conduct delinquent acts (Rebellon 2002).

Similar to the case of economic disadvantage, research on the effect of family intactness has mainly been conducted to examine the level of delinquent behavior (e.g., Jolliffe 2013; Shek and Leung 2013), while much remains unknown about how it affects developmental trajectory. For an exception, VanderValk et al. (2005) investigated the trajectories of externalizing behavior problems including delinquent behavior in 12–24 year olds. They found that although more externalizing behavior problems were observed in youngsters from divorced families as compared to those from intact families over time, the developmental patterns over 3 years were similar regardless of family structure. However, it is still possible that similar to poor families, non-intact families could not provide adequate social resources and support to help adolescents conquer their challenges in the transition or control their increasing delinquent impulsivity. In this case, the growth of delinquent behavior in adolescents from non-intact families might be greater. Thus, more research is needed to identify whether the growth rate of delinquent behavior varies by family intactness.

### Protective Factors: Family Functioning and Positive Youth Development

Despite the heightened level of delinquent behavior during adolescence, previous research has suggested that healthy family functioning and positive youth development are linked to a lower level of delinquent behavior. Family functioning pertains to the quality of family life as a whole, including yet beyond the dyadic relationship (Shek 2002a, b). While healthy family functioning is characterized by strong connectedness, open communication, and mutuality from a Western perspective (Quatman 1997), strong connectedness, mutuality, absence of conflicts, interpersonal harmony, and positive parent–child relationship are intrinsic to the Chinese culture (Shek 2002a).

Does family functioning matter on adolescent delinquent behavior? It is argued that the impacts of family functioning factors decline over the adolescent period due to the rising counterinfluence of peer group (Reitz et al. 2006). Through modelling and reinforcement, association with deviant peers is often regarded as a major cause of adolescent problem behavior (e.g., Ary et al. 1999; Fergusson et al. 2002). Adolescents with friends having higher levels of delinquent behavior are more likely to engage in delinquent behavior (e.g., Brendgen et al. 2000). In some studies, peer group factors outperformed family functioning factors when predicting adolescent delinquent behavior (e.g., Ary et al. 1999; Reitz et al. 2006).

Nonetheless, there are also many studies indicating that family factors remain significant for the behavioral adjustment of teenagers (e.g., Buehler 2006; Galambos et al. 2003). Maladaptive family environment may have a unique contribution to the adolescent delinquent behavior above and beyond the peer group influence (e.g., Buehler 2006). Furthermore, it may increase the opportunity of associating with deviant peer, which further increases the delinquent acts of the adolescents (e.g., Kim et al. 1999). According to the social control perspective (Hirschi 1969), better perceived family functioning helps to shape a stronger bonding with family, which prevents adolescents from delinquent involvement (Hoeve et al. 2012). In particular, a warm and harmonious family climate facilitates adolescents’ internalization of conventional norms expected by parents, which possibly reduces the likelihood to engage in norm-breaking and risk behavior even without the presence of parents. Furthermore, effective communication and mutual support among family members enable parents to sanction offspring’s problem behavior timely. To illustrate, open communication enables parents to obtain knowledge of adolescents, inform them the appropriateness of behavior, and regulate their behavior accordingly (Pardini et al. 2008).

Regarding personal well-being, the construct of positive youth development is derived from the positive youth development perspective (Benson et al. 2006; Lerner et al. 2012; Roth and Brooks-Gunn 2003) which maintains that all youth have personal strengths or potential to be developed. Rather than focusing on managing youth problems, this perspective advocates optimizing youth positive functioning. It maintains that fostering adolescents’ myriad developmental assets in individual and context could enhance the likelihood of thriving and lessen the likelihood of problem behavior (Benson et al. 2006; Lerner et al. 2012). These developmental assets serve to buffer life stress during adolescence, which possibly renders adolescents less likely to externalize their stress to delinquent behavior (Shek et al. 2011a). By reviewing 25 high-quality positive youth development programs in the United States, Catalano et al. (2004) revealed that 96 % of the programs showed effectiveness in minimizing problem behavior. This is also the case in Hong Kong, where the large-scale project (i.e., Positive Adolescent Training through Holistic Social Programmes; Project P.A.T.H.S.) conducted to nurture Hong Kong adolescents’ developmental assets has shown to reduce the growth of delinquent behavior during adolescence (Catalano et al. 2012; Shek and Yu 2012).

Empirically speaking, results based on the cross-sectional research are roughly consistent, with family functioning and positive youth development being negatively associated with delinquent behavior. As to family functioning, Delsing et al. (2005) showed that the general levels of perceived justice and trust among family members negatively predicted adolescents’ levels of problem behavior. Another study in Hong Kong (Shek 2002b) also showed that good family functioning characteristic of higher level of communication and support while lower level of conflict at the systematic level was associated with lower level of delinquent behavior.

As to positive youth development, its inverse association with adolescent problem behavior was exemplified in Geldhof et al.’s (2014) study based on eight waves of data (Grade 5–12) when it was measured in terms of 5Cs model—competence, confidence, character, caring, and connection (Lerner et al. 2012; Roth and Brooks-Gunn 2003). Additionally, it was observed in a Hong Kong study on 7975 Secondary 1 students (Sun and Shek 2010) and replicated with these students 1 year later (Sun and Shek 2012), when it was assessed in terms of Catalano et al. (2004)’s proposed 15 developmental assets—bonding, resilience, cognitive competence, emotional competence, social competence, behavioral competence, moral competence, self-determination, self-efficacy, clear and positive identity, belief in the future, spirituality, development of prosocial norms, opportunities for prosocial involvement, and recognition for positive behavior.

Nevertheless, there remains a large knowledge gap about the longitudinal effects of family functioning and positive youth development on adolescent delinquency. Theoretically, good family quality of life and positive youth development protect adolescents from vulnerability to risks and lay the groundwork for long-term adolescent behavioral adjustment (Benson et al. 2006; Lerner et al. 2012; Marsiglia et al. 2009; Pettit et al. 1997; Sun and Shek 2013). However, there are two unresolved issues. For the one thing, similar to the case of risk factors, scanty research has investigated how early family functioning and positive youth development predict the change of delinquent behavior over the adolescent years. Yet a few studies have suggested that better individual functioning and family functioning predict the adaptive trajectory of behavioral adjustment in adolescence. To illustrate, Hoeve et al. (2008)’s study revealed that neglectful parenting was related to up-and-down and serious persisting pattern of delinquency while authoritarian parenting was related to serious persisting pattern of delinquency compared to nondelinquent trajectory. Galambos et al. (2003)’s study found that parental behavioral control was associated with a smaller increase of problem behavior. These studies, however, focused on the dyadic level of family functioning alone, which might not be able to fully capture the quality of family life at a systematic level. Additionally, in the Project P.A.T.H.S., researchers (Shek and Yu 2012) found that participating students demonstrated a slower increase of delinquent behavior than did their control counterparts without receiving treatment. This study, however, did not assess positive youth development directly.

Besides, the direction of effect represented by the association remains unclear given that most of the research to date is cross-sectional. Sun and Shek (2012) raised the concern that cross-sectional research could not exclude the alternative explanation that adolescent problem dampens positive youth development. This possibility is also applicable to the relationship between family functioning and adolescent delinquent behavior (Shek 2005). The longitudinal relationship between family functioning and delinquent behavior has been seldom documented in the extant literature. One exception conducted by Shek and Lin (2014a) found 1-year and 2-year prediction of family functioning on adolescent delinquent behavior, and the other conducted by Shek (2005) found longitudinal effect of family functioning for females (not males) but he only studied poor adolescents. The longitudinal relationship between positive youth development and adolescent problem behavior documented in previous literature was not always inverse as theoretically assumed. Using the first two waves of data from the 4-H Study of Positive Youth Development, Jelicic et al. (2007) found that positive youth development in Grade 5 predicted a lower level of problem behavior (i.e., substance use and delinquency) in Grade 6. However, Lewin-Bizan et al. (2010a)’s study using four waves of data (Grade 5–8) in this project revealed that early positive youth development did not predict later delinquent behavior. Similarly, Shek and Lin (2014a) did not find the long-term prediction of positive youth development.

Against this background, we investigated not only the concurrent associations of these protective factors and delinquent behavior but also the long-term prediction of these protective factors in this study. The outcomes of interest include both the level of delinquent behavior and developmental course of delinquent behavior. First, many cross-sectional studies were conducted in early adolescence (Shek 2005; Shek and Lin 2014a; Sun and Shek 2010, 2012), with the links unclear in middle adolescence. Thus we could use the assessment of Secondary 4 students to test whether family functioning and positive youth development are inversely associated with delinquency when adolescents grow older. Second, considering the scanty and mixed results of the longitudinal effects of family functioning and positive youth development on the level of delinquent behavior, we examined them in this study. It would be also intriguing to explore whether their predictive power would remain over a longer period of time (i.e., 3 years). Lastly, we had an additional interest about how family functioning and positive youth development in the initial level predict the developmental course of delinquent behavior in subsequent years.

### Current Research

The primary objective of the current research was to understand adolescent delinquent behavior from a developmental perspective. To achieve this objective, we assessed a large sample of Hong Kong Chinese adolescents over 4 years approximately annually. With individual growth curve, we first examined the shape of the growth curve of delinquent behavior with an expectation of an increasing trend over 4 years (Hypothesis 1).

Next, we particularly investigated whether economic disadvantage (i.e., receiving Comprehensive Social Security Assistance or not) and family intactness (after controlling age and gender) would be associated with the initial level and growth rate of delinquent behavior (i.e., how fast the delinquent behavior increased or decreased over time). On the basis of the literature described above (e.g., Lansford 2009; Mcloyd et al. 2009), we generally hypothesized that adolescents growing up in families with adversity would show more delinquent behavior. Specifically, we sought to test two hypotheses on the initial level.

#### **Hypothesis 2a:**

Adolescents from poor families would have higher initial levels of delinquent behavior than those from non-poor families.

#### **Hypothesis 2b:**

Adolescents from non-intact families would have higher initial levels of delinquent behavior than those from intact families.

Given a dearth of evidence reporting how the change of delinquent behavior differs among adolescents from different families, we explored whether the shape of the growth curve of delinquent behavior differs according to economic disadvantage and family intactness. In the individual growth curve models, we controlled initial age and gender, as previous research has indicated that male and older adolescents are more likely to conduct delinquent behavior than did female and younger adolescents (e.g., Farrell et al. 2005; Overbeek et al. 2001; Shek and Yu 2012). However, effects of initial age and gender on the developmental trajectories of delinquent behavior were open for exploration because of the limited and inconsistent results (Overbeek et al. 2001; Shek and Yu 2012).

Furthermore, we examined the effects of family functioning and positive youth development on the initial level and growth rate of delinquent behavior beyond the effects of demographic factors. Two additional hypotheses (Hypothesis 3a and 3b) were presented below based on prior research findings (e.g., Jelicic et al. 2007; Shek 2005). Similar to the cases of demographic factors, we explored the relationship of delinquent behavior trajectory with family functioning and positive youth development without specific hypotheses.

#### **Hypothesis 3a:**

Adolescents with better family functioning have lower initial levels of delinquent behavior relative to those with worse family functioning.

#### **Hypothesis 3b:**

Adolescents with better positive youth development have lower initial levels of delinquent behavior relative to those with worse positive youth development.

Next, we examined whether the risk factors (i.e., economic disadvantage and family non-intactness) and protective factors (i.e., family functioning and positive youth development) would predict delinquent behavior concurrently (Wave 4) and longitudinally (Wave 1 variables predicting Wave 4 delinquent behavior). Based on previous literature documenting the harmful impacts of economic disadvantage and family non-intactness (e.g., Lansford 2009; Mcloyd et al. 2009) and desirable impacts of family functioning and positive youth development on the level of delinquent behavior (e.g., Jelicic et al. 2007; Shek 2005; Shek and Lin 2014a; Sun and Shek 2012), we sought to test four additional hypotheses for their concurrent effects (Hypothesis 4a, 4b, 4c, 4d) and four for their longitudinal effects (Hypothesis 5a, 5b, 5c, 5d):

#### **Hypothesis 4a:**

Economic disadvantage would be positively associated with delinquent behavior at Wave 4;

#### **Hypothesis 4b:**

Family non-intactness would be positively associated with delinquent behavior at Wave 4;

#### **Hypothesis 4c:**

Family functioning would be negatively associated with delinquent behavior at Wave 4;

#### **Hypothesis 4d:**

Positive youth development would be negatively associated with delinquent behavior at Wave 4;

#### **Hypothesis 5a:**

Economic disadvantage at Wave 1 would predict increased delinquent behavior at Wave 4;

#### **Hypothesis 5b:**

Family non-intactness at Wave 1 would predict increased delinquent behavior at Wave 4.

#### **Hypothesis 5c:**

Family functioning at Wave 1 would predict declined delinquent behavior at Wave 4;

#### **Hypothesis 5d:**

Positive youth development at Wave 1 would predict declined delinquent behavior at Wave 4.

## Methods

### Participants and Procedure

The current study included four assessment waves with approximately 1-year intervals, which was drawn from an on-going 6-year longitudinal study. We recruited students from 28 secondary schools randomly selected from all the Government and Aided secondary schools in Hong Kong. At Wave 1, the sample consisted of 3328 adolescents between 10 and 18 years old (Mage = 12.59 ± 0.74 years; 47.2 % female) from Secondary 1. Parental consent and school consent were obtained before Wave 1 assessment. Student individual consent was also obtained before administration of each wave of assessment. Therefore, there were new participants who volunteered to join the assessment after Wave 1. For the students taking the Wave 1 assessment, most of them continued to join the subsequent assessments, with an attrition rate of 12.7 % (Wave 2), 14.1 % (Wave 3) and 19.4 % (Wave 4). The characteristics of participants are presented in Table 1.

**Table 1:** Data profile across four waves

	Wave 1	%	Wave 2	%	Wave 2^a^	%	Wave 3	%	Wave 3^a^	%	Wave 4	%	Wave 4^a^	%
*N* (participants)	3328		3638		2905		4106		2858		3973		2682	
Gender														
Male	1719	51.7	1716	47.2	1445	49.7	1885	45.9	1433	50.1	1875	47.2	1335	49.8
Female	1572	47.2	1864	51.2	1419	48.8	2185	53.2	1405	49.2	2086	52.5	1337	49.9
Economic disadvantage														
NOT receiving CSSA	2606	78.3	2932	80.6	2377	81.8	3308	80.6	2339	81.8	3302	83.1	2267	84.5
Receiving CSSA	225	6.8	208	5.7	160	5.5	212	5.2	147	5.1	200	5.0	132	4.9
Family intactness														
Intact families	2781	83.6	2985	82.1	2415	83.1	3372	82.1	2396	83.8	3210	80.8	2211	82.4
Non-intact families	515	15.5	624	17.2	469	16.1	715	17.4	454	15.9	749	18.9	466	17.4
Divorced but not remarried	209	6.3	256	7.0	199	6.9	345	8.4	207	7.2	350	8.8	206	7.7
Separated but not remarried	73	2.2	78	2.1	62	2.1	95	2.3	67	2.3	96	2.4	68	2.5
Remarried	129	3.9	168	4.6	116	4.0	189	4.6	122	4.3	205	5.2	125	4.7
Others	104	3.1	122	3.4	9.2	3.2	86	2.1	5.8	2.0	98	2.5	67	2.5

^a^The numbers were based on the participants who ever participated in Wave 1 assessment as only those joining Wave 1 assessment were included in LMM. The numbers of the students who did not report the corresponding information were not presented

Students completed a battery of questionnaires, which included their delinquent behavior, family functioning, positive youth development and other demographic information, during regular school hours in a classroom setting. A trained research was present during the assessment and emphasized the study purpose and the confidentiality of the data to all the participants.

### Instruments

#### Delinquent Behavior

The delinquent behavior scale is comprised of 12 delinquent acts: stealing, cheating, truancy, running away from home, damaging others’ properties, assault, having sexual intercourse with others, gang fighting, speaking foul language, staying outside the home overnight without parental consent, strong arming others, and trespassing behavior. Adolescents indicated how often they engaged in these delinquent acts in the past year on a 7-point scale (0 = never, 1 = one to two times; 2 = three to four times; 3 = five to six times; 4 = seven to eight times; 5 = nine to ten times; 6 = more than ten times). Items were averaged to indicate the overall level of adolescents’ delinquent engagement. The reliabilities across the four waves were satisfactory, *α*s > .69.

#### Family Functioning as an Indicator of Family Quality of Life

Adolescents reported on their perceived family functioning by the abbreviated version of the Chinese Family Assessment Instrument with nine items on a 5-point scale (1 = very dissimilar; 5 = very similar; Shek 2002a). Three facets of family functioning were assessed, which are mutuality (mutual support, love, and concern among family members), communication (frequency and nature of interaction among family members), conflicts and harmony (presence of conflicts and harmonious behavior in the family). After reversing the items of family conflicts, items were averaged to indicate the family quality of life. Higher numbers were indicative of better family functioning. Four waves of assessments demonstrated good reliabilities, *α*s > .90.

#### Positive Youth Development as an Indicator of Personal Well-Being

Adolescents reported on their positive youth development with the trimmed version of the Chinese Positive Youth Development Scale (CPYDS; Shek et al. 2007; Sun and Shek 2010). The CPYDS was developed on the basis of developmental assets proposed by Catalano et al. (2004), which include bonding, resilience, social competence, recognition for positive behavior, emotional competence, cognitive competence, behavioral competence, moral competence, self-determination, self-efficacy, clear and positive identity, beliefs in the future, prosocial involvement, prosocial norms, and spirituality. Each subscale of developmental asset includes two to three items, with a response format ranging from 1 = strongly disagree to 6 = strongly agree, except spirituality (7-point scale). Items were averaged to indicate the overall positive youth development. The measure was reliable across four waves, *α*s > .96.

#### Family Attributes: Economic Disadvantage and Family Intactness

Economic disadvantage was categorized in terms of receiving Comprehensive Social Security Assistance (CSSA) or not. In Hong Kong, CSSA was given to families which had financial difficulties, and has been proved to indicate poor economic well-being (Wong 2005). Thus, we used it as a symbol of poverty in the current study. At the initial assessment, among all the participating adolescents, 225 (6.8 %) students reporting receiving CSSA were categorized as having economic disadvantage; while 2606 (78.3 %) students reporting not receiving CSSA were categorized as not having economic disadvantage (see Table 1).

Family intactness was indicated by one item about marital status of adolescents’ parents (1 = divorced but not remarried, 2 = separated but not remarried, 3 = married (first marriage), 4 = remarried, 5 = others). Adolescents who indicated “first marriage” were categorized as living in intact families, while those who indicated other options were categorized as living in non-intact families. At the initial assessment, among all the participating adolescents, 2781 (83.6 %) adolescents were identified as living in intact families, while 515 (15.5 %) adolescents were identified as living in non-intact families (see Table 1). Adolescents who did not report aforementioned information were not included in corresponding analyses using the information.

### Data Analyses Plan

Four questions of our main interest include: (1) developmental trajectory of delinquent behavior; (2) the effects of economic disadvantage and family intactness on the initial status (i.e., Wave 1) and change rate of delinquent behavior; (3) the effects of family functioning and positive youth development on the initial status and change rate of delinquent behavior; (4) concurrent and longitudinal impacts of economic disadvantage, family intactness, family functioning, and positive youth development on adolescent delinquent behavior.

To begin with, individual growth curve modeling (i.e., longitudinal multilevel analyses) was employed to address questions 1 to 3. Individual growth curve can estimate the individual changes over time and examine the effects of individual differences on the initial status and growth rate, which has been commonly used in tracking adolescent development (e.g., Farrell et al. 2005; Shek and Yu 2012; VanderValk et al. 2005). In our study, a two-level hierarchical model that nested time (Level 1) within individual (Level 2) was created. Time was coded as 0 = Wave 1, 1 = Wave 2, 2 = Wave 3, 3 = Wave 4. The first level deals with the repeated measures, describing the normative developmental trajectory, including the average within-person initial status and rate of change over time without other predictors involved. The second level taps into the individuals, exploring how individual characteristics affect the initial status and the rate of change of delinquent behavior. We primarily examined the effects of economic disadvantage, family intactness, family functioning and positive youth development with gender and initial age controlled.

We conducted individual growth curve modeling via four steps. First, we estimated unconditional mean model (Model 1) to establish how much of the variance of delinquent behavior could be found at the different levels (i.e., intraclass correlation coefficient or ICC; Singer and Willett 2003). This model did not involve any predictors. Second, we estimated an unconditional growth model (Model 2), in which the pattern of change over time was examined. Given only four waves of assessment available, linear change was examined for getting a stable trend. Third, we estimated the conditional model with economic disadvantage and family intactness as major predictors while initial age and gender were controlled (Model 3). Finally, we estimated the conditional model with family functioning and positive youth development as major predictors while other demographic factors were controlled (Model 4). For the level-2 predictors, categorical variables were dummy-coded (gender: female = −1; male = 1; economic disadvantage: not receiving CSSA = −1; receiving CSSA = 1; family intactness: non-intact family = −1; intact family = 1), and continuous variables (i.e., initial age, family functioning and positive youth development) were grand-mean centered in order to simplify the interpretation of the results (Shek and Ma 2011). The proposed final models for delinquent behavior, denoted by the term, *Y*_*ij*_, were as follows:

Level 1: *Y*_*ij*_ = *β*_0*j*_ + *β*_1*j*_ (Time) + *r*_*ij*_

where *β*_0*j*_ is the initial status of delinquent behavior for individual *j*, *β*_1*j*_ is the rate of change for individual *j; r*_*ij*_ is the residual in the delinquent behavior for individual *j* at Time *i*, and *Y*_*ij*_ is the repeated measure of delinquent behavior for an individual *j* at Time *i.*

Level 2: *β*_0*j*_ = *γ*_00_ + *γ*_01_ (age) + *γ*_02_ (gender) + *γ*_03_ (economic disadvantage) + *γ*_04_ (family intactness) + *γ*_05_ (family functioning) + *γ*_06 _(positive youth development) + *u*_0*j*_
*β*_1*j*_ = *γ*_10_ + *γ*_11_ (age) + *γ*_12_ (gender) + *γ*_13_ (economic disadvantage) + *γ*_14_ (family intactness) + *γ*_15_ (family functioning) + *γ*_16 _(positive youth development) + *u*_1*j*_

where *γ*_01_, *γ*_02_, *γ*_03_, *γ*_04_, *γ*_05_, *γ*_06_ are used to test whether the factors are associated with the initial status of delinquent behavior; *γ*_11_, *γ*_12_, *γ*_13_, *γ*_14_, *γ*_15_, *γ*_16_ are used to test the extent to which the developmental change of delinquent behavior varies as a function of the factors; *γ*_00_ is the level of delinquent behavior when the values of predictors are equal to zero. *γ*_10_ is the linear slope of change relating to the delinquent behavior when the values of predictors are equal to zero; *u*_0*j*_ and *u*_1*j*_ are the residuals that are not explained by level-2 predictors for the intercept and slope, respectively.

For evaluating model, we referred to three indexes that have been commonly used in previous research: −2log likelihood (i.e., likelihood ratio test), Akaike information criterion (AIC), and Bayesian information criterion (BIC) (Shek and Ma 2011; Shek and Yu 2012; Wray-Lake et al. 2010). Smaller numbers indicate better model fit. Linear mixed model (LMM) in SPSS 21.0 statistical software (IBM SPSS Statistics, IBM Corp., Somers, NY, USA) was used to perform individual growth curve with maximum likelihood (ML) as estimation method. Only participants who joined the Wave 1 assessment were retained in the individual growth curve as they reported initial information serving as level-2 predictors (*N* = 3328).

Our final interest regards the concurrent and over-time effects of these risk factors and protective factors on the level of delinquent behavior (question 4), which was addressed by multiple regression analyses. For concurrent effects, demographic variables were entered into the regression model at the first step, and family functioning and positive youth development were added into the regression model at the second step. For over-time effects, initial level of delinquent behavior was entered into the regression model at the first step, demographic variables were then added at the second step, and family functioning and positive youth development were finally added at the third step.

## Results

### Delinquency Behavior Across Time

As shown in Tables 2 and 3, across 4 years of secondary school, the occurrence of delinquent behavior among Hong Kong adolescents was quite low, with the percentages of most delinquent behavior <10 %. However, over this period, speaking foul language and cheating were relatively popular among Hong Kong adolescents, approximately 70 and 60 % of the adolescents reported ever speaking foul language and cheating, respectively. Furthermore, quite a significant proportion of adolescents reported ever damaging others’ properties and engaging in assault (>10 %). From Table 3, we could observe an increase in the mean level of delinquent behavior with time. Further analyses were conducted to examine the developmental pattern.

**Table 2:** Descriptive statistics of key variables and internal consistency coefficients of scales (Waves 1–4)

	Mean (SD)				Reliability			
	Wave 1	Wave 2	Wave 3	Wave 4	Wave 1	Wave 2	Wave 3	Wave 4
Family functioning	3.73 (.81)	3.65 (.81)	3.65 (.79)	3.66 (.77)	.90	.90	.90	.91
Positive youth development	4.51 (.70)	4.43 (.69)	4.44 (.65)	4.45 (.62)	.96	.96	.96	.96
Delinquent behavior								
Overall	.39 (.47)	.47 (.58)	.46 (.55)	.48 (.54)	.70	.76	.72	.69
Male	.43 (.51)	.52 (.60)	.52 (.60)	.55 (.60)	–	–	–	–
Female	.35 (.42)	.43 (.55)	.39 (.46)	.39 (.46)	–	–	–	–
NOT receiving CSSA	.39 (.46)	.47 (.56)	.46 (.51)	.47 (.52)	–	–	–	–
Receiving CSSA	.41 (.42)	.53 (.63)	.50 (.63)	.56 (.61)	–	–	–	–
Intact families	.37 (.46)	.45 (.57)	.43 (.48)	.46 (.54)	–	–	–	–
Non-intact families	.48 (.52)	.56 (.60)	.60 (.78)	.52 (.56)	–	–	–	–
Divorced but not remarried	.44 (.47)	.57 (.66)	.50 (.50)	.47 (.48)	–	–	–	–
Separated but not remarried	.44 (.37)	.54 (.50)	.60 (.73)	.51 (.45)	–	–	–	–
Remarried	.51 (.52)	.51 (.42)	.53 (.47)	.51 (.47)	–	–	–	–
Others	.53 (.66)	.57 (.54)	.54 (.44)	.63 (.60)	–	–	–	–

**Table 3:** Percentages of participants engaging in different types of delinquent behavior across four waves

	Never				Attempted 1–6 times				Attempted more than 6 times			
	Wave 1	Wave 2	Wave 3	Wave 4	Wave 1	Wave 2	Wave 3	Wave 4	Wave 1	Wave 2	Wave 3	Wave 4
1. Stealing	2980 (89.6)	3273 (90.0)	3662 (89.2)	3614 (91.0)	318 (9.6)	318 (8.7)	284 (6.9)	200 (5.0)	16 (0.5)	41 (1.1)	27 (0.7)	22 (0.6)
2. Cheating	1298 (39.0)	1378 (37.9)	1644 (40.0)	1615 (40.6)	1602 (48.2)	1678 (46.1)	1625 (39.6)	1496 (37.7)	410 (12.3)	565 (15.5)	690 (16.8)	722 (18.2)
3. Truancy	3205 (96.4)	3438 (94.5)	3731 (90.9)	3602 (90.7)	96 (2.9)	164 (4.5)	210 (5.1)	197 (5.0)	13 (0.4)	30 (0.8)	28 (0.7)	37 (0.9)
4. Running away from home	3182 (95.7)	3455 (95.0)	3784 (92.2)	3715 (93.5)	127 (3.8)	161 (4.4)	174 (4.2)	113 (2.8)	5 (0.2)	11 (0.3)	13 (0.3)	10 (0.3)
5. Damaging others’ properties	2861 (86.0)	3164 (87.0)	3593 (87.5)	3513 (88.4)	417 (12.5)	419 (11.5)	337 (8.2)	280 (7.0)	27 (0.8)	46 (1.3)	38 (0.9)	41 (1.0)
6. Assault	2922 (87.9)	3226 (88.7)	3633 (88.5)	3605 (90.7)	342 (10.3)	341 (9.4)	276 (6.7)	178 (4.5)	46 (1.4)	60 (1.6)	58 (1.4)	46 (1.2)
7. Sexual intercourse	3290 (98.9)	3572 (98.2)	3903 (95.1)	3742 (94.2)	20 (0.6)	37 (1.0)	40 (1.0)	55 (1.4)	2 (0.1)	17 (0.5)	29 (0.7)	39 (1.0)
8. Gang fighting	3182 (95.7)	3477 (95.6)	3841 (93.5)	3724 (93.7)	96 (2.9)	103 (2.8)	88 (2.1)	66 (1.7)	14 (0.4)	30 (0.8)	26 (0.6)	32 (0.8)
9. Speaking foul language	1000 (30.1)	1041 (28.6)	1259 (30.7)	1196 (30.1)	1458 (43.8)	1335 (36.7)	1234 (30.1)	1074 (27.0)	802 (24.1)	1244 (34.2)	1459 (35.5)	1550 (39.0)
10. Staying outside overnight without parents’ consent	3177 (95.5)	3462 (95.2)	3772 (91.9)	3628 (91.3)	80 (2.4)	124 (3.4)	142 (3.5)	145 (3.6)	19 (0.6)	34 (0.9)	42 (1.0)	43 (1.1)
11. Strong arming others	2762 (83.1)	3082 (84.7)	3545 (86.3)	3480 (87.6)	436 (13.1)	435 (12.0)	319 (7.8)	263 (6.6)	77 (2.3)	111 (3.1)	102 (2.5)	91 (2.4)
12. Trespasses	3139 (94.4)	3501 (96.2)	3853 (93.8)	3738 (94.1)	112 (3.4)	102 (2.8)	87 (2.1)	72 (1.8)	11 (0.3)	19 (0.5)	29 (0.7)	25 (0.6)

0 = never, 1–3 = attempted 1–6 times, 4–6 = attempted more than 6 times

As the original scales of delinquent behavior were positively skewed (skewness between 1.89 and 2.72; kurtosis between 6.97 and 16.77), we performed log-transformation for the overall scores of delinquent behavior. After log-transformation, skewness and kurtosis fell within acceptable ranges (skewness between 0.75 and 1.26; kurtosis between 0.22 and 1.83). Accordingly, log-transformed scores were used for further analyses.

Individual growth curve modeling was performed to examine (1) the trajectory of adolescent delinquent behavior; (2) the effects of economic disadvantage and family intactness on the initial status and growth rate of delinquent behavior with initial age and gender controlled; (3) the effects of family functioning and positive youth development on the initial status and growth rate of delinquent behavior with the demographic factors controlled. The results of models were presented in Table 4. First, unconditional mean model (Model 1) suggested that about 57.1 % of the total variation in the delinquent behavior was due to inter-individual differences (ICC = .571), which indicated a need for further multi-level analyses (Lee 2000; Shek and Ma 2011).

**Table 4:** Results of individual growth curve of delinquent behavior for all models

		Model 1		Model 2		Model 3		Model 4	
		Estimate	SE	Estimate	SE	Estimate	SE	Estimate	SE
**Fixed effects**									
*Intercept*	*β*_0*j*_								
Initial status	*γ*_00_	.322***	.004	.298***	.005	.324***	.010	.302***	.010
Age	*γ*_01_					.022**	.007	.015*	.008
Gender^a^	*γ*_02_					.018**	.005	.016*	.005
Economic^b^ disadvantage	*γ*_03_					−.002	.011	−.008	.011
Family intactness^c^	*γ*_04_					−.032***	.008	−.007	.008
Family functioning	*γ*_05_							−.082***	.008
Positive youth development	*γ*_06_							−.086***	.009
*Linear slope*	*β*_1*j*_								
Initial status	*γ*_10_			.018***	.002	.026***	.004	.029***	.004
Age	*γ*_11_					−.006*	.003	−.008*	.003
Gender^a^	*γ*_12_					.012***	.002	.013***	.002
Economic^b^ disadvantage	*γ*_13_					.009*	.004	.009	.005
Family intactness^c^	*γ*_14_					.003	.003	−.002	.003
Family functioning	*γ*_15_							.013***	.003
Positive youth development	*γ*_16_							.008*	.004
**Random effects**									
Level 1 (within)									
Residual	*r*_*ij*_	.0370***	.001	.0316***	.001	.0314***	.0314	.0313***	.001
Level 2 (between)									
Intercept	*u*_0*j*_	.0493***	.002	.0504***	.002	.0489***	.0489	.0356***	.002
Time	*u*_1*j*_			.0029***	.000	.0028***	.0028	.0024***	.0003
**Fit statistics**									
Deviance		400.247		137.567		−51.541		−551.983	
AIC		406.247		149.567		−23.541		−515.983	
BIC		428.206		193.486		76.482		−391.387	
df		3		6		14		18	

Model 1 = unconditional mean model; Model 2 = unconditional growth model; Model 3 = conditional model

^a^Male = 1, Female = −1; ^b^ Receiving CSSA = 1, Not receiving CSSA = −1; ^c^ Intact = 1, Non-Intact = −1

*** *p* < .001; ** *p* < .01; * *p* < .05

Second, unconditional growth model (Model 2) showed a better model fit than Model 1 (*χ*_(3)_^2^ = 262.68; *p* < .001; ΔAIC = 256.68; ΔBIC = 234.72). According to Model 2, adolescents’ delinquent behavior increased linearly at a slow rate. Hence, Hypothesis 1 was supported. The results of random effects showed that participants varied significantly both in their intercepts and linear slopes, which indicated that level-2 predictors can be included to examine the inter-individual differences in the intercepts and linear slopes.

Third, with the level-2 risk predictors, the conditional model (Model 3) demonstrated a better model fit than Model 2 (Δχ_(8)_^2^ = 189.11; *p* < .001; ΔAIC = 173.11; ΔBIC = 117.00). For the predictors of the intercept, results showed that family intactness was negatively associated with the initial level of delinquent behavior above and beyond the effects of initial age and gender. Specifically, adolescents from non-intact families showed more delinquent acts than those from intact-families at the initial assessment, which supported Hypothesis 2b. However, economic disadvantage was not related to the initial status of delinquent behavior, which indicated delinquent behavior at the initial assessment did not vary according to the receipt of CSSA. Hence, Hypothesis 2a was not supported. Level-2 predictors explained 3.0 % of the variance of intercept.

For the predictors of slope, results revealed that economic disadvantage was positively associated with the growth rate of delinquent behavior above and beyond the effects of initial age and gender. Specifically, adolescents from poor families increased delinquent behavior faster than those from non-poor families. Nevertheless, family intactness was not linked to the rate of change, which indicated that the developmental pattern of delinquent behavior did not differ between adolescents from intact and non-intact families. Level-2 predictors explained 4.51 % of the variance of the slope. Yet supplemental analyses showed that adolescents living in non-intact families showed higher levels of delinquent behavior in all four waves [Wave 1: *t*(3087) = 4.91, *p* < .001; Wave 2: *t*(3496) = 4.77, *p* < .001; Wave 3: *t*(3844) = 6.58, *p* < .001; Wave 4: *t*(3725) = 3.05, *p* < .01]. These results suggested that family intactness was still a risk factor.

Finally, when family functioning and positive youth development were entered into the LMM model as level-2 predictors (Model 4), the model fitted the data better compared with Model 3 [Δχ_(4)_^2^ = 500.44; *p* < .001; ΔAIC = 492.44; ΔBIC = 467.87]. As expected (Hypotheses 3a and 3b), initial levels of family functioning and positive youth development were negatively associated with the initial level of delinquent behavior above and beyond the effects of demographic factors. Yet, they were positively associated with the growth rate of delinquent behavior above and beyond the effects of demographic factors. Specifically, the better the initial family functioning or positive youth development, the faster the increase of delinquent behavior. However, suggested by the gamma coefficients, the faster increase in the adolescents with better family functioning or positive youth development did not offset the higher initial level of delinquent behavior among those with worse family functioning or positive youth development. Noteworthy, with the inclusion of these two psychosocial factors, the effects of economic disadvantage and family intactness became insignificant.

**Fig. 1:**
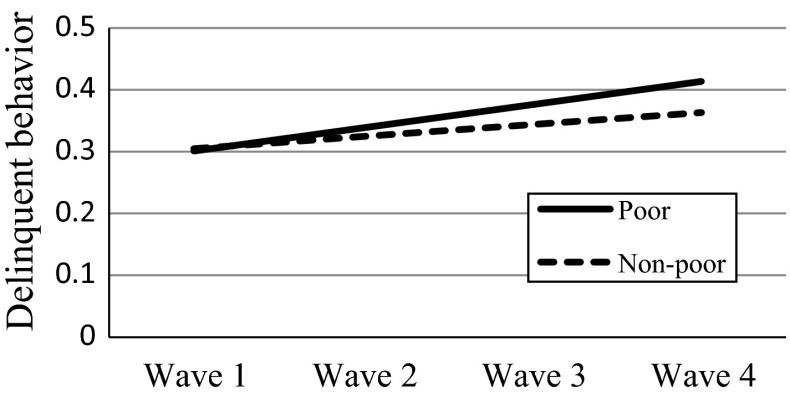
Effect of economic disadvantage on the slope of delinquent behavior. *Note*: the plot was based on the results of Model 3

**Fig. 2:**
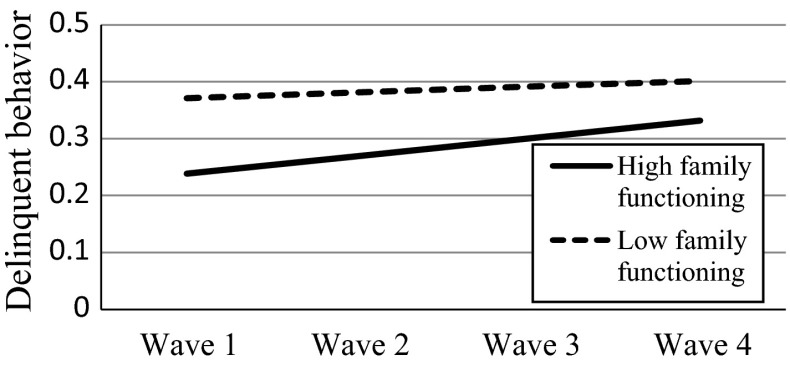
Effect of family functioning on the slope of delinquent behavior. *Note*: the plot was based on the results of Model 4

**Fig. 3:**
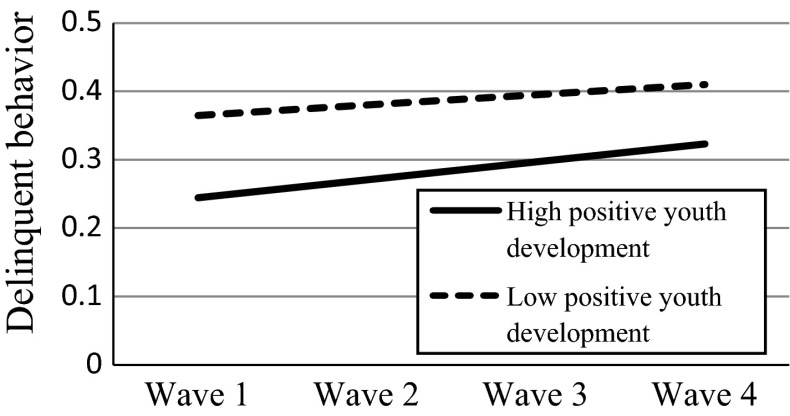
Effect of positive youth development on the slope of delinquent behavior. *Note*: the plot was based on the results of Model 4

To demonstrate the effects of level-2 predictors on the slope of delinquent behavior, we plotted the effects by substituting the prototypical values into the equations (Aiken and West 1991; Singer and Willett 2003). For the effect of economic disadvantage, we obtained the fitted trajectories by substituting two values (1 = poor; −1 = non-poor) based on Model 3 (see Fig. 1). For the effects of family functioning and positive youth development, we substituted two commonly used values: one standard deviation above the mean value of the predictor and one standard deviation below the mean value of the predictor based on Model 4 (see Figs. 2, 3). The figures also showed that despite the faster increase among the adolescents with higher initial levels of family functioning and positive youth development, these adolescents still reported less delinquent behavior compared with those with lower initial levels.

### Concurrent and Longitudinal Effects of Family Functioning and Positive Youth Development

For concurrent effects (see Table 5), economic disadvantage and family intactness were not significant, which did not support Hypotheses 4a and 4b. Beyond the effects of demographic factors, family functioning and positive youth development were both inversely related to delinquent behavior, which added to explaining 8.5 % of the total variance, which supported Hypotheses 4c and 4d. These findings indicated that mid-adolescents with better family functioning or positive youth development demonstrated lower levels of delinquent behavior.

**Table 5:** Multiple regression analyses on delinquent behavior at Wave 4

Predictors	Beta	R^2^ change
Step 1		.033***
Gender^a^	.169***	
Economic disadvantage^b^	.034	
Family intactness^c^	−.009	
Step 2		.085***
Family functioning	−.157***	
Positive youth development	−.188***	

*** *p* < .001

^a^Male = 1, female = 0; ^b^ receiving CSSA = 1, not receiving CSSA = 0; ^c^ intact = 1, non-intact = 0

Out of expectation (Hypotheses 5a, 5b, 5c, 5d), none of the risk factors and protective factors had longitudinal effects when the initial level of delinquent behavior was controlled (see Table 6). A supplemental analysis without controlling the initial level of delinquent behavior revealed that Wave 1 family functioning (*β* = −.081, *p* < .01) and positive youth development (*β* = −.137, *p* < .001) significantly predicted Wave 4 delinquent behavior. Yet there were no long-term predictive effects of the risk factors.

**Table 6:** Wave 1 variables predict Wave 4 delinquent behavior

Predictors	Beta	R^2^ change
Step 1		.213***
Initial delinquent behavior	.439***	
Step 2		.029***
Gender^a^	.162***	
Economic disadvantage^b^	.024	
Family intactness^c^	−.030	
Step 3		.001
Family functioning	.010	
Positive youth development	−.035	

^a^Male = 1, female = 0; ^b^ receiving CSSA = 1, not receiving CSSA = 0; ^c^ intact = 1, non-intact = 0

*** *p* < .001

## Discussion

In response to the call for viewing adolescent delinquent behavior with a developmental perspective (Bongers et al. 2004), the current study investigated the relationship of delinquent behavior with economic disadvantage, family intactness, family functioning and positive youth development via a longitudinal research design. Going beyond the cross-sectional “snapshot” of adolescent delinquent behavior, our study examined the developmental trajectories, individual differences of economic disadvantage, family intactness, family functioning and positive youth development in developmental trajectories, as well as their longitudinal effects on the level of delinquent behavior. The findings of the study are generally in line with the theories, although some findings are at odds with the original expectations. In short, this study gives new insights to our understanding of adolescent delinquent behavior based on data collected from Chinese adolescents.

Consistent with the expectation, delinquent behavior increased during secondary school years. In conjunction with previous literature showing a rise of problem behavior during early adolescence (e.g., Bongers et al. 2003; Deković et al. 2004; Shek and Yu 2012), this study implies that such an escalation also exists in Chinese adolescents. Accordingly, early adolescence can be regarded as a critical period for the intervention of delinquent behavior.

In addition to the normative developmental pattern, this study advances our understanding of individual differences in this pattern. Previous literature has suggested that economic disadvantage is a risk factor for adolescent delinquency (McLoyd et al. 2009), while our study further suggests that such risk may not only be manifested in the occurrence but also in the developmental pattern. Specifically, notwithstanding no difference in the initial level of delinquent behavior between poor adolescents and non-poor adolescents, poor adolescents seem to increase delinquent behavior involvement faster than non-poor adolescents.

As we speculated, poor adolescents without sufficient resource, support and experience from family may be more vulnerable to developmental challenges that render them involved in delinquent behavior. The impact of economic disadvantage on the change rate of delinquent behavior, notwithstanding small, implies the possibility that the developmental patterns of poor adolescents might deviate from those non-poor adolescents. Yet whether such a greater increase will maintain over a longer period among poor adolescents is still unknown. It would be intriguing to examine how poverty relates to developmental trajectory of delinquency throughout late adolescence and early adulthood, when a decline is expected to occur (Overbeek et al. 2001).

Meanwhile, we did not find that family intactness influenced the developmental pattern of delinquent behavior, though it was linked to the initial level of delinquent behavior. Similar findings were shown by VanderValk et al. (2005). It is possible that deviant change of delinquent behavior simply occurs within a short period after family restructuring. Tracing trajectories of externalizing behavior over time, Malone et al. (2004)’s study revealed a rise in adolescent males’ but not adolescent females’ externalizing behaviors in the year of the divorce. However, such behavioral problems declined in the subsequent years of divorce. Thus, future studies contrasting adolescents within the critical period of family restructuring and those in intact families are sorely needed.

However, even if family intactness was not associated with developmental pattern of delinquent behavior, adolescents living in non-intact families consistently demonstrated higher levels of delinquent behavior than those living in intact families across four waves of data. Therefore, attention is still needed for this group of adolescents given their heightened baseline of delinquent behavior. Furthermore, it is also too early to refute the impact of non-intact families on adolescents’ developmental pattern due to different types of living arrangements in non-intact families. The risks may vary across different types of non-intact families and adolescents in them may experience distinct developmental patterns of psychological adjustment (Coleman et al. 2000; Jeynes 2006). On the one hand, adolescents in single-parent families may have less financial and parental resource than do their counterparts in remarried families due to absence of parent. On the other hand, adolescents in remarried families may encounter greater stresses in getting along with their new family members. In addition, it is also controversial whether single-mother families differ from single-father families. Some scholars argued that female-headed families are generally at a greater risk, such as poverty (e.g., Buvinic and Gupta 1997), while others maintained that female headship is not necessarily associated with more problems (e.g., Bradshaw and Quirós 2008). Instead, prior research suggests that the risk of behavioral problem may be greater among adolescents in single-father families than their counterparts with other living arrangements (e.g., Breivik and Olweus 2006). Owing to the limited sample size in each type of non-intact family (see Table 1) in the current study, we were unable to explore the potential differentiated development patterns just mentioned. Obviously, future studies on this topic would benefit from a scrutiny on specific types of non-intact family structure.

On the other hand, we shall interpret the unique effects of risk factors with caution since these effects became insignificant with the effects of psychosocial factors (i.e., family functioning and positive youth development) included in the model. Similar insignificant unique prediction could be found in the regression models. It is possible that adverse family status is translated into maladaptive adolescent functioning through family functioning and positive youth development, while it awaits to be validated by more studies. These results also indicate that the objective indicators of adverse family status might be less important than quality of family life and personal well-being processes perceived by the adolescents. Results are consistent with the previous findings that structural family factors (i.e., SES and family structure) were less significant than proximate family factors (e.g., quality of parent–child relationship) in predicting adolescent problematic behavior (Deković et al. 2003). This is a piece of good news since the latter psychosocial factors are malleable, evidenced by the previous intervention and prevention programs (Santisteban et al. 2003; Shek and Ma 2011).

Congruent with previous cross-sectional studies (e.g., Geldhof et al. 2014; Shek 2002b), the current study found that family functioning and positive youth development were negatively associated with the level of delinquent behavior concurrently. These findings suggest that they are protective factors that minimize the likelihood of adolescent delinquent involvement. Nevertheless, their long-term predictions in the developmental trajectory and level of delinquent behavior are out of our expectation.

Despite the assumption that better family functioning and positive youth development lay a constructive groundwork for adolescent behavioral adjustment, the current study demonstrated some complex effects. Out of expectation, they predicted faster increase of delinquent behavior. This finding echoes an interesting argument in the emerging literature that better-functioning adolescents “experiment” on risk behavior (e.g., Arbeit et al. 2014; Lewin-Bizan et al. 2010b), while such an experimentation might be developmentally appropriate and even constructive for identity formation (Dworkin 2005). For example, Arbeit et al. (2014)’s study found that the high level of competence and confidence was not only associated with the low risk behavior profile but also the profile characteristic of engagement in some aggressive behavior and drug use. Furthermore, what kind of risk behavior these well-functioning adolescents attempt is still a question. In Arbeit et al. (2014)’s study, adolescents who were categorized as low risk group were more likely to engage in sexual activity with protection while those in high risk group more likely to try unprotected sexual activity. While engagement in unprotected/unwanted sexual activity is problematic, it is natural that engagement in sexual activity is growing in adolescence. Actually, in the current study, the better-functioning adolescents did not report more delinquent behavior than the worse-functioning adolescents despite a slightly faster increase. Lower levels of family functioning and positive youth development were still indicative of a higher level of delinquent behavior. Yet the current findings are far from conclusive. With more waves of assessments, future studies may be able to testify whether such a faster growth reflects an “experiment” that is only specific to adolescence. We also need more support from studies examining other adolescent samples and other problem behavior (e.g., substance use).

Furthermore, out of expectation, the longitudinal effects of family functioning on the overall delinquent behavior were not significant in our study. Combining with the results in Shek and Lin (2014a)’s study finding that Wave 1 family functioning can predict Wave 2 and Wave 3 adolescent adjustment, it is possible that the long-term effect of family functioning might be minimized over time. Many other factors that become increasingly important in adolescents’ lives, such as school climate and peer group (Dishion et al. 1991; Reitz et al. 2006), may intervene into the over-time effect of family functioning during the process.

The weak prediction of family functioning and positive youth development might be due to two other possibilities regarding the sample and method. Firstly, adolescents dropped out in Wave 4 assessment reported higher initial level of delinquent behavior than adolescents joining both Wave 1 and Wave 4 assessments: *t*(3114) = 7.84, *p* < .001. With the dropout of these participants, the over-time change of delinquent behavior observed might become smaller, which possibly reduced the longitudinal effects. Because young people can work at the age of 15 in Hong Kong, students who do not perform well in school may drop out to work. Secondly, we used a stringent longitudinal approach by adjusting the initial level of delinquent behavior, whereas the initial level was not controlled in Jelicic et al. (2007)’s study that revealed the significant effects of positive youth development. If the initial level of delinquent behavior was not controlled, Wave 1 family functioning and positive youth development were significantly associated with Wave 4 delinquent behavior.

Several major contributions of this study should be noted. To begin with, given the large randomly selected sample across Hong Kong, the developmental trajectories obtained in this study can be regarded as a normative description of delinquent behavior from early to middle adolescence for Chinese adolescents in Hong Kong. The development of delinquent behavior in high-risk groups may be contrasted with the present normative profile.

Secondly, the results of individual differences in developmental trajectories (together with effects of initial age and gender that were controlled in the model) inform us that risk and protective factors can influence the developmental pattern of adolescent adjustment in addition to the level of adolescent adjustment at a single point. For the majority of the adolescents, the escalation of delinquent behavior often occurs only in adolescence, while a few persist in the course of life (i.e., adolescence-limited vs. life-course-persistent; Moffitt 1993). Probing into the individual differences in developmental trajectories of delinquent behavior allows us to know more about why some people demonstrate rapidly increasing or enduring delinquent behavior throughout adolescence, and provide evidence-based intervention accordingly (Deković et al. 2004). Therefore, other factors documented in prior research, including neighborhood poverty and disorganization (Murry et al. 2011), school disorder (Gottfredson et al. 2005) and peer relationship (Dishion et al. 1991), could be testified in relation to developmental pattern of delinquent behavior as well. Yet it does not mean that their effects on the level of delinquent behavior are not important, as they both provide information about how risk and protective factors affect adolescent adjustment.

Finally, together with other emerging studies, the current findings point to the revision of models to understand how family attributes, family quality of life and personal well-being impinge on adolescent behavior. Taking positive youth development model as an example, is positive development always linked to decreased risk behavior? Evidence that seems counterintuitive is mounting (e.g., Arbeit et al. 2014; Lewin-Bizan et al. 2010b). The major proposition could be maintained, given that most of the empirical findings (e.g., concurrent associations) are in line with the theory. Meanwhile, there is a need for efforts at the revision of theory and ideas for application given the long-term effects. The results suggest that the theory about the inverse relation between positive development and negative behavior is not a must. Instead, it is possibly constrained by certain conditions, which requires further investigation. Also, if positive youth development could not predict adolescent risk behavior over a 3-year interval, one-shot programs promoting positive youth development might be not effective to prevent adolescents from delinquent involvement. Continuous effort at the promotion of positive youth development becomes necessary. The success of Project P.A.T.H.S. (Shek et al. 2011b; Shek and Yu 2012) might be due to the multi-year design.

This study is not without limitations. While we viewed delinquent behavior with a developmental perspective, we tested risk factors and protective factors as static factors in this study. However, it is possible that people move in and out of poverty over time (Mistry et al. 2002), and other factors also could be varying during the assessment period (e.g., family functioning, Cordova et al. 2014; positive youth development; Lewin-Bizan et al. 2010b). The lack of inquiry into such dynamics may be one of the reasons for weak longitudinal effects observed in this study. Therefore, tracking how changes of economic status, family structure, family functioning and positive youth development relate to development of delinquent behavior could be the next step for researchers to take. For example, we may categorize economic disadvantage status and family disruption by its duration (Pagani et al. 1999). We could also test how developmental profiles of delinquent behavior relate to the trajectory of positive youth development (Arbeit et al. 2014; Lewin-Bizan et al. 2010b) and change of family functioning.

Another limitation of this study is the reliance on only self-reports to assess delinquent behavior. Adolescents might under-report their delinquent behavior due to social desirability and peer pressure, as they completed the questionnaire in a classroom setting with other participants. Therefore, replications with parental report, teacher report or peer report are highly encouraged to validate the developmental patterns (Bongers et al. 2003). Primarily, convergent evidence is needed by using multiple informants. Besides, adolescent delinquent behavior is possibly situation-specific (Moffitt 1993), and thus variations in results are expected due to different informants.

The third limitation regards the measures of positive youth development and delinquent behavior. The positive youth development perspective suggests that youth thriving is comprised of healthy and flourishing growth of multiple attributes (Lerner et al. 2010). We tested an arguable hypothesis of positive youth development that youth thriving is indicative of fewer problem behaviors (e.g., Lerner et al. 2010) in this research. However, because of the use of overall score to indicate positive youth development rather than the scores of different components, this research can only shed light on how overall thriving impacts on delinquent behavior. Similarly, we used the overall score of delinquent behavior, which was also applied in previous studies (e.g., Deković et al. 2003; Farrell et al. 2005). Yet it limits our conclusion to the overall delinquent level. To further probe into why the relation between positive youth development and problem behavior is not perfectly inverse, future studies might explore if different components of positive youth development bear distinct implications for reducing different delinquent acts. As to the practical application, Project P.A.T.H.S. including 120 teaching units with reference to 15 components of positive youth development (Shek et al. 2011b) have demonstrated the benefits of promoting these 15 components (Catalano et al. 2012; Shek and Yu 2012). Yet in order to provide further implication for prevention program, it is necessary to dig up which component will be more effective for minimizing delinquent involvement in the future (Shek and Sun 2013).

The fourth limitation is the lack of examination on peer group influence in our study although it is well-documented in the literature that peer has a unique effect on adolescent delinquent involvement (e.g., Ary et al. 1999; Reitz et al. 2006). Additionally, the interplay of family and peer group is also salient in early adolescence (Lansford et al. 2003; Vitaro et al. 2000). As such, further studies should be conducted to examine how developmental patterns of delinquent behavior vary according to the family and peer factors simultaneously (see Galambos et al. 2003).

Lastly, the use of CSSA as an indicator of economic disadvantages limits the representativeness of poor adolescents. The adolescents who have no idea of whether their families are receiving CSSA and those who are poor but have not obtained this subsidy (such as the working poor or poor parents refusing to rely on welfare) would be excluded from the investigation. For an accurate objective index of economic status, parent-report family income would be more encouraged in the future studies. Yet such information is usually confidential information that the parents do not want to disclose. As the Hong Kong Government has released the first official poverty line recently (Ip 2013), more studies are required to testify if people living under poverty line suffer from poor psychosocial quality of life in addition to economic well-being (Wong 2005), and what factors may help poor adolescents in holistic development.
